# Tackling Chronic Kidney Transplant Rejection: Challenges and Promises

**DOI:** 10.3389/fimmu.2021.661643

**Published:** 2021-05-20

**Authors:** Xingqiang Lai, Xin Zheng, James M. Mathew, Lorenzo Gallon, Joseph R. Leventhal, Zheng Jenny Zhang

**Affiliations:** ^1^ Comprehensive Transplant Center, Northwestern University Feinberg School of Medicine, Chicago, IL, United States; ^2^ Department of Surgery, Northwestern University Feinberg School of Medicine, Chicago, IL, United States; ^3^ Organ Transplant Center, the Second Affiliated Hospital of Guangzhou Medical University, Guangzhou, China; ^4^ Department of Urology, Beijing Youan Hospital, Capital Medical University, Beijing, China; ^5^ Department of Medicine, Nephrology, Northwestern University Feinberg School of Medicine, Chicago, IL, United States

**Keywords:** chronic allograft rejection, kidney transplant, biomarkers, IFTA, T cells mediated rejection

## Abstract

Despite advances in post-transplant management, the long-term survival rate of kidney grafts and patients has not improved as approximately forty percent of transplants fails within ten years after transplantation. Both immunologic and non-immunologic factors contribute to late allograft loss. Chronic kidney transplant rejection (CKTR) is often clinically silent yet progressive allogeneic immune process that leads to cumulative graft injury, deterioration of graft function. Chronic active T cell mediated rejection (TCMR) and chronic active antibody-mediated rejection (ABMR) are classified as two principal subtypes of CKTR. While significant improvements have been made towards a better understanding of cellular and molecular mechanisms and diagnostic classifications of CKTR, lack of early detection, differential diagnosis and effective therapies continue to pose major challenges for long-term management. Recent development of high throughput cellular and molecular biotechnologies has allowed rapid development of new biomarkers associated with chronic renal injury, which not only provide insight into pathogenesis of chronic rejection but also allow for early detection. In parallel, several novel therapeutic strategies have emerged which may hold great promise for improvement of long-term graft and patient survival. With a brief overview of current understanding of pathogenesis, standard diagnosis and challenges in the context of CKTR, this mini-review aims to provide updates and insights into the latest development of promising novel biomarkers for diagnosis and novel therapeutic interventions to prevent and treat CKTR.

## Introduction

Chronic kidney transplant rejection (CKTR) is characterized by progressive decrease of renal graft function that starts to manifest at one-year after the transplantation and usually accompanied by hypertension and proteinuria (1). CKTR usually occurs in patients with insufficient immunosuppression or medication nonadherence (2). While Persistent allogeneic immune response remains a major cause (3, 4), multiple risk factors, e.g. early ischemia reperfusion injury, acute rejection episodes and transplant infectious diseases, can contribute to the development and progression of CKTR. Histologically, there are two principal distinct subtypes of CKTR, namely chronic active antibody-mediated rejection (ABMR) and chronic active T cell-mediated rejection (TCMR) according to the revised Banff criteria (5, 6). It is not uncommon that both chronic active TCMR/ABMR co-exist and lead to rapid loss of graft function (7–9).

Effective treatment and prognosis of CKTR are largely dependent upon the severity and reversibility of rejection at the time of diagnosis. However, it remains a major challenge to identify early changes before irreversible damage to the graft occurs. Currently, no immunotherapies are clinically proven to be effective in prevention and treatment of CKTR, particularly ABMR. Recent advances in high-throughput cellular and molecular biotechnologies have allowed for in-depth analyses of cellular and molecular processes and deconvolutions of mechanisms underlying CKTR and have led to identification and validation of new molecular and cellular biomarkers through non-invasive or minimal-invasive approaches. The discovery of these biomarkers holds tremendous promise for early detection and development of promising novel therapies for improvement of kidney transplant outcomes. This review will first provide a brief summary on current understanding of pathogenesis and standard mothed challenges for the diagnosis of CKTR, and then, focus on more in-depth discussions to the area of biomarker discovery and novel therapeutic interventions to improve long term transplant outcome.

## Pathogenesis of Chronic Active ABMR and Chronic TCMR

Chronic active ABMR represents most cases of CKTR (2), featuring transplant glomerulopathy along with severe peritubular capillary basement membrane multilayering and new onset arterial intimal fibrosis. In contract, chronic active TCMR is determined based on inflammation in areas of the cortex with interstitial fibrosis and tubular atrophy (i-IFTA), a hallmark feature of CKTR in addition to tubulitis. The newly revised Banff criteria of chronic active TCMR recognize the pathogenic importance of TCMR in the development of chronic interstitial inflammation leading to i-IFTA, nonetheless, it does not discriminate alloimmune-mediated tissue injury from ‘non-specific injuries, particularly calcineuirn inhibitor (CNI) mediated nephrotoxicities (10, 11).

While precise mechanisms underlying ABMR remain elusive, it is believed that the interaction of donor-specific alloantibodies (DSAs) against donor HLA antigens, especially HLA class II antigens expressed by endothelial cells of the microvascular circulation, initiates ABMR (12). DSAs binding to endothelial cells leads to a cascade of molecular events, including complement activation that may contribute to endothelial dysfunction, microvascular inflammation and remodeling, and ultimately results in irreversible tissue injury (13). B cell deficiency resulted in reduced transplant glomerulopathy, decreased microvascular inflammation, reduced macrophage infiltration and IFNγ transcripts in the allograft (14), which underscores the importance of B cells in the pathogenesis of ABMR. In addition to uncontrolled allogeneic immune response due to insufficient immunosuppression or nonadherence, early inflammatory events such as acute TCMR and viral infection are suggested to be risk factors for DSA (dnDSA) production (15–17). Preceding TCMR is found to be strongly correlated with development of chronic active ABMR dnDSA (7). Moreover, it has been shown in biopsy-proven chronic active ABMR cases that T cells (especially CD8+ T cells) and macrophages are the dominant infiltrating cell types in glomerulus, whereas B cells are frequently observed in the tubulointerstitial compartment, indicating that both T cells and macrophages play a pivotal role in renal chronic ABMR (18). The involvement of NK cells in ABMR has recently gained attention. Recent studies have revealed that NK cells are involved in ABMR *via* CD16a Fc receptors (19, 20). Depletion of NK cells significantly mitigates DSA-induced chronic allograft vasculopathy (CAV) (21). NK cells increase IFNγ production after exposure to alloantigens through an antibody-dependent cellular cytotoxicity-like mechanisms, which is associated with an increased risk for ABMR (22) and NK cell infiltration predicts poor outcome after kidney transplantation (23).

Persistent T cell–mediated injuries can lead to chronic active TCMR (24). Alloreactive effector memory T Cells (Tem), particularly CD8+ Tem subsets (express increased CD44hi, CD45RO+, OX40, KLRG-1 and BLIMP-1), are implicated in the development of TCMR (25). Unlike naïve T cells, Tem cells are known for their low activation threshold, robust effector functions, and resistance to conventional immunosuppression and costimulation blockade (26). Memory T cells are originated from environmental antigens or generated from previous rejection episodes and once activated, they enter into the renal interstitium and secrete several cytokines such as IFNγ and TGFβ, and subsequently trigger a cascade of inflammation leading to tubulitis (27). Chronic TCMR also results in renal vasculature injuries, such as arterial inflammation and intimal fibrosis (6). In a recent study, Claudia and colleagues (25) demonstrated that CD8+ effector memory T cells mediated by the OX40 gene pathway play an important role in the pathogenesis of chronic TCMR.

## Current Diagnosis and Challenges

Early diagnosis of CKTR determines successful therapeutic interventions and prognosis. CKTR is a slowly progressive process in which pathologic changes as such vascular inflammation and i-IFTA do not have clinical manifestations until late stages. In addition, differential diagnosis is extremely important to distinguish CKTR from late graft dysfunction caused by other complications including CNI toxicity, BK-virus associated nephropathy and recurrent renal diseases, each of which requires different treatment. Transplant patients are subjected to routine laboratory tests for continuous graft monitoring. Serum creatinine (sCr), blood urea nitrogen (BUN) and cystatin C are commonly used to evaluate graft function. The estimated glomerular filtration rate (eGFR), calculated based on sCr level, age, weight and gender, is considered as an accurate indicator and predictor for graft function and long-term graft survival (28). Proteinuria>500 mg/day is also considered as a marker of chronic kidney allograft dysfunction (29). However, because chronic rejection is an indolent process with slow progression in pathologic changes (30), these aforementioned tests are non-specific, often failing to detect renal damage at early stages and easily influenced by other non-immune injuries can also influence the results. Emergence of circulating *de novo* DSAs is associated with increased risk for graft failure as a result of chronic active ABMR (31, 32). Prospective monitoring for DSAs may be indicative for early treatment before irreversible graft injury (33, 34), however, not all DSAs are doomed to be pathogenic (35) and DSA levels may not correlate with tissue injury (15). Imaging technologies such as Doppler ultrasonography (US), Contrast-enhanced ultrasound (CEUS) and Magnetic Resonance Imaging (MRI) are non-invasive complementary methodologies used to assist in the early diagnosis of both acute and chronic graft rejection by evaluating renal vasculature resistance (US) (36, 37), graft blood perfusion (CEUS) (38), and anatomical changes (MRI) such as fibrosis (39). However, findings from these tests are mostly non-specific with limited value in guiding the clinical treatment.

Currently, graft biopsies still remain the gold standard for diagnosing graft rejection. Graft histology provides visual evidence of the underlying pathology and pathogenesis of graft dysfunction. More recently, genetic analysis of biopsy tissue has been used to assist in the differential diagnosis of allograft rejection in conjunction with histology and immunohistochemistry. The Banff classification, founded in 1991, has established specific criteria for the diagnosis of kidney allograft rejection. It has been updated multiple times in the past two decades (5). C4d complement fragment deposition in the peritubular capillaries was regarded as a marker for ABMR (40), but removed as a diagnostic criterion in the latest Banff (2019) Classification Criteria for chronic active ABMR due to the emergence of C4d negative ABMR (41). Although histological examination through renal biopsy remains the diagnostic gold standard criterion, it cannot be carried out too often due to its invasiveness. Graft needle biopsy can cause various surgery-related complications, such as perinephric hematoma, arteriovenous fistula, bleeding, infection. In addition, there are other limitations associated with histologic examination, e.g. lack of standardization and quantitation, sampling errors and accurate diagnosis largely relies on the pathologists’ skills (42). Therefore, non-/minimally-invasive and predictive biomarkers are highly desirable for early diagnosis and tailored inventions to delay or prevent CKTR and improve graft longevity.

## Potential Biomarkers for Early Diagnosis and Prognosis

Recent development of high-throughput cellular and molecular biotechnologies has led to tremendous advances in biomarker discoveries in the field of transplantation, with great promise for better understanding and management of CKTR. Contributions of biomarker studies are multifold, including 1) generating new insight into molecular mechanisms of CKTR, 2) allowing for early and differential diagnosis, 3) providing evaluation of therapeutic intervention, and 4) predicting prognosis. Principal characteristics of the biomarkers have been thoroughly reviewed elsewhere (42–44). Although most studies have centered in exploring non-invasive biomarkers for ischemia/reperfusion injury and acute allograft rejection in blood and urine (42), a variety of biomarkers generated from studies in renal protocol biopsies and blood and urine samples are suggestive for diagnosis and prognosticator for CKTR. Based on the characteristics of the biomarkers and technologies used, biomarkers pertaining to CKTR can be divided into five main categories: transcriptomic biomarkers, Epigenetic biomarkers, Proteomic biomarkers, and Metabolomic biomarkers, and cellular biomarkers, which are summarized **Table 1**, and also briefly discussed in the following sections.

**Table 1:** Potential biomarkers for chronic rejection.

Biomarker classification	Biomarker candidate	Sample type	AUC	Sensitivity/Specificity	Application	Ref
Transcriptomic biomarker	CD6, INPP5D, ISG20, NKG7, PSMB9, RUNX3, TAP1 (↑)	Kidney graft	n/a	n/a	Predict the development of progressive i-IFTA at 24 months	Sigdel TK, et al. (45)
	vimentin, NKCC2, E-cadherin, 18S rRNA (↑)	Urine	0.95	0.938/0.841	As a 4-gene model diagnostic of i-IFTA	Lee JR, et al. (46)
	CHCHD10, KLHL13, FJX1, MET, SERINC5, RNF149, SPRY4, TGIF1, KAAG1, ST5, WNT9A, ASB15, RXRA	Kidney graft	0.889 (average)	0.81/0.79 (average)	Predict fibrosis and graft failure	O’Connell PJ, et al. (47)
	RSAD2, ETV7 (↑)	PBMC	0.761 (RSAD2) 0.84 (ETV7)	n/a	Diagnose of ABMR	Matz M, et al. (48)
	TIM-3 (↑)	PBMC	0.71	0.83/0.75	Predict CAD	Shahbaz SK, et al. (49)
		Urine	0.75	0.83/0.75		
	TIM-3, KIM-1 (↑)	PBMC	0.99 (TIM-3)	1/0.98 (TIM-3)	Predict CAD	Shahbaz SK, et al. (50)
			0.97 (KIM-1)	1/0.7 (KIM-1)		
		Urine	0.95 (TIM-3)	1/0.81 (TIM-3)		
			0.99 (KIM-1)	1/0.93 (KIM-1)		
			0.95 (KIM-1 concentration)	1/74 (KIM-1 concentration)		
	CIITA (↓), CTLA-4 (↑)	PBMC	0.902 (CIITA)	n/a	Predict dnDSA and chronic ABMR	Yamamoto T, et al. (51)
			0.785 (CTLA4)			
	TLR-2, TLR-4, MyD88 (↑)	PBMC	0.94 (TLR2)	0.93/0.93 (TLR2)	Predict early and late CAD	Hosseinzadeh M, et al. (52)
			0.95 (TLR4)	0.93/0.93 (TLR4)		
			0.94 (MyD88)	1/0.93 (MyD88)		
		Kidney graft	0.94 (TLR2)	0.93/0.93 (TLR2)		
			0.95 (TLR4)	0.93/1 (TLR4)		
			0.98 (MyD88)	1/0.93 (MyD88)		
	CASP3, FAS, IL-18 (↓)	PBMC	0.79 (CASP3)	0.71/0.88 (CASP3)	Predict graft function	Kaminska D, et al. (53)
			0.75 (FAS)	0.64/0.8 (FAS)		
			0.77 (IL-18)	0.71/0.8 (IL-18)		
Epigenetic biomarkers	Foxp3 DNA demethylation	Kidney graft	n/a	n/a	Protector for long-term allograft outcome	Bestard O, et al. (54)
	PD1 DNA methylation in memory CD8+ T cells (↑)	PBMC	n/a	n/a	PD1 DNA methylation increases in recipients with rejection	Karin Boer, et al. (55)
	miR-21, miR-200b (↑)	Urine	0.89 (miR-21)	0.85/0.8 (miR-21)	Corelate with renal allograft dysfunction and i-IFTA; diagnostic biomarkers for renal allograft monitoring	Zununi VS, et al. (56)
			0.81 (miR-200b)	0.84/0.95 (miR-200b)		
	miR-150 (↑), miR-423-3p (↑), miR192 (↓), miR-200b (↓)	Plasma	0.87 (all)	0.78/0.91	Predict graft outcome in recipients with CAD	Zununi VS, et al. (57)
	miR21, miR-155, miR-142-3p (↑)	Plasma	0.82 (all)	0.81/0.92	Upregulate in recipients with i-IFTA; corelate with renal allograft dysfunction; can be used for graft monitoring	Zununi VS, et al. (58)
	miR-145-5p (↓)	Plasma	0.891	0.933/0.731	Diagnostic biomarker of i-IFTA	Matz M, e al (59)
	miR-148a (↓)	Plasma	0.89	0.97/0.72	Correlated with renal function and histological grades; biomarker of the progression to i-IFTA	Nariman-Saleh-Fam Z, et al. (60)
	miR-142-3p(↓), miR-204 (↑), miR-211 (↑)	Urine, kidney graft	0.974 (miR-142-3p)	0.89/1 (miR-142-3p)	As markers of CAD with i-IFTA and for monitoring graft function	Scian MJ, et al. (61)
			0.967 (miR-204)	0.95/1 (miR-204)		
			1 (miR-211)	1/1 (miR-211)		
	miR-142-5p (↓), miR-486-5p (↑)	PBMC	n/a	n/a	Predict chronic ABMR	Iwasaki K, et al. (62)
Proteomic biomarker	V305_HUMAN_NTLYLNMNSLR, RL18_HUMAN_ILTFDQLALDSPK, F151A_HUMAN_AVGPSLDLLR, TGFR2_HUMAN_LTAQCVAER, LYAM1_HUMAN_AEIEYLEK, K2C8_HUMAN_LSELEAALQR, F151A_HUMAN_TYTQAMVEK, PLGB_Human_AFQYHSK, K1C19_HUMAN_ILGATIENSR, IBP7_HUMAN_GTCEQGPSIVTPPK, LV102_HUMAN_WYQQLPGTAPK DSRAD_Human_YLNTNPVGGLLEYAR,	Urine	0.995	n/a	Predict CAD	Sigdel TK, et al. (63)
	PARP1 (↓)	Serum	0.871	n/a	Predict AR and chronic graft injury	Srivastava M, et al. (64)
	TNF-α, ANXA11, Integrin α3, Integrin β3 (↑)	Urine	0.805 (TNFα)	n/a	Diagnose AR and CR	Srivastava M, et al. (65)
			0.855 (Integrin α3)			
			0.813 (integrin β3)			
			0.963 (ANXA11)			
	CXCL9, CXCL10 (↑) CXCL9/Cr ratio (↑) CXCL10/Cr ratio (↑)	Urine	0.86 (CXCL9), 0.9 (CXCL9/Cr)	n/a	Predict TCMR	Rabant M, et al. (66)
			0.8 (CXCL10), 0.82 (CXCL10/Cr)	n/a	Predict mixed rejection	
			0.7 (CXCL10), 0.7 (CXCL10/Cr)	n/a	Predict ABMR	
	CXCL10/Cr ratio (↑)	Urine	0.81 (sub-clinical TCMR)	0.59/0.67 (subclinical TCMR)	Predict TCMR for pediatric recipients	Blydt-Hansen TD, et al. (67)
			0.88 (clinical TCMR)	0.77/0.6 (clinical TCMR)		
	Vitronectin (↑)	Urine	0.963	n/a	Monitor fibrotic changes in kidney allograft	Carreras-Planella L, et al. (68)
	Properdin, sC5b-9 (↑)	Urine	n/a	n/a	As risk factors of graft failure	Lammerts R, et al. (69)
	AZGP1 (↑)	Urine	0.946	0.846/0.8	Predict and diagnose chronic ABMR	Jung HY, et al. (70)
	β2 microglobulin, NGAL, clusterin, KIM-1 (↑)	Urine	n/a	n/a	Predict chronic allograft nephropathy	Cassidy H, et al. (71)
Metabolomic biomarkers	Newly Synthesized DNA and ATP	PBMC	n/a	n/a	Analyze lymphocyte subset activation responses	Sottong PR, et al. (72)
	NAD, 1-MN, cholesterol sulfate, GABA, nicotinic acid, NADPH, proline, spermidine, alpha-hydroxyhippuric acid	Urine	n/a	n/a	Predict TCMR	Kalantari S, et al. (73)
	Alanine, Citrate, Lactate, combined with urea or glucose or glucutonate	Urine	0.76	n/a	Diagnose AR	Miriam B, et al. (74)
	threitol, inositol, glucose, xylono-1, 5-lactone, xylitol, xylopyranoside, 2,3-dihydroxybutanoic acid, glucitol, ribonic acid, octadecanoic acid, phosphate (↑) fructose, glycolic acid, 3-hydroxyisovaleric acid (↓)	Urine	n/a	0.867/0.677	Diagnose AR	Long Zheng, et al. (75)
	guanidoacetic acid, methylimidazoleacetic acid, dopamine (↑) 4-guanidinobutyric acid, L-tryptophan (↓)	Urine	0.926	0.9/0.846	Diagnose AR	Kim S, et al. (76)
	Itaconate, kynurenine (↑)	Kidney graft	n/a	n/a	Distinguish acute cellular rejection from IRI	Beier UH, et al. (77)
	glycine, glutaric acid, adipic acid, inulobiose, threose, sulfuric acid, taurine, *N*-methylalanine, asparagine, 5-aminovaleric acid lactam, myo-inositol	Urine	0.985	0.929/0.963	Diagnose AR	Sigdel TK, et al. (78)
Cellular biomarker	TEMRA/EM CD8 T cell ratio (↑)	PBMC	0.75 (8 year graft failure)	n/a	Predict graft failure	Jacquemont L, et al. (79)
			0.79 (11 year graft failure)			
	CD154+ T-cytotoxic memory cells (↑)	PBMC	0.968	0.923/0.846	Predict rejections (liver)	Ashokkumar C, et al. (80)
		PBMC	0.938	1/0.88	Predict AR (kidney)	Ashokkumar C, et al. (81)
	alloreactive memory IFN-γ-producing T cells (↑)	PBMC	0.725	0.8/0.64	Predict subclinical TCMR and DSA	Crespo E, et al. (82)
	Ratio of T follicular helper cells and T follicular regulatory cells (Tfc/Tfr) (↑)	PBMC	n/a	n/a	Risk factor of CAD	Yan L, et al. (83)
	Myofibroblast	Kidney graft	n/a	n/a	Identify CR	Liu YG, et al. (84)

NKCC2, Na-K-Cl cotransporter 2; CD6, cluster of differentiation 6; INPP5D, inositol polyphosphate-5-phosphatase D; ISG20, interferon-stimulated gene 20; NKG7, natural killer cell granule protein 7; PSMB9, proteasome subunit beta type-9; RUNX3, runt-related transcription factor 3; TAP1, transporter associated with antigen processing 1; CHCHD10, Coiled-coil-helix-coiled-coil-helix domain containing 10; KLHL13, Kelch-like family member 13; FJX1, Four jointed box 1; MET, Met proto-oncogene; SERINC5, Serine incorporator 5; RNF149, Ring finger protein 149; SPRY4, Sprouty homolog 4; TGIF1, TGFB-induced factor homeobox 1; KAAG1, Kidney associated antigen 1; ST5, Suppression of tumorigenicity 5; WNT9A, Wingless-type MMTV integration site family member 9A; ASB15, Ankyrin repeat and SOCS box-containing 15; RXRA, Retinoid X receptor alpha; TIM-3, T cell immunoglobulin and mucin domain 3; KIM-1, kidney injury molecule-1; CIITA, class II transactivator; CTLA-4, cytotoxic T-lymphocyte antigen; TLR, toll-like receptor; MyD88, myeloid differentiation factor 88; CASP3, caspase 3; FAS, first apoptotic signal; PD1, programmed death 1; miR, micro RNA; PARP1, Poly(ADP-ribose) polymerase 1; CXCL9, chemokine C-X-C motif ligand 9; CXCL10, chemokine C-X-C motif ligand 10; AZGP1, zinc-alpha-2-glycoprotein; NAD, nicotinamide adenine dinucleotide; 1-MN, 1-methylnicotinamide; GABA, gamma-aminobutyric acid; NADPH, nicotinamide adenine dinucleotide phosphate; IRI, ischemia reperfusion injury; NGAL, neutrophil gelatinase-associated lipocalin; TEMRA, terminally differentiated effector memory; EM, effector memory; PBMC, Peripheral blood mononuclear cell; AUC, area under curve; n/a, not available; I-IFTA, interstitial fibrosis and tubular atrophy; AR, acute rejection; CR, chronic rejection; ABMR, antibody-mediated rejection; TCMR, T cell-mediated rejection; CAD, chronic allograft dysfunction; dnDSA, de novo donor specific antibody.

### Transcriptomic Biomarkers

These biomarkers are generated by high-throughput gene or transcriptome profiling, also termed transcriptomics, using microarray and next generation gene sequencing technologies. These studies have been more commonly performed on renal biopsy samples as they provide sufficient material for RNA extraction. As listed in **Table 1**, gene signatures associated with fibrosis, i-IFTA, chronic rejection (ABMR and TCMR) and graft failure can be identified by determining gene expression profiling (45–53). Importantly, the gene set has higher predictive capacity than that of baseline clinical variables, and clinical and pathological variables. One notion from these studies is that similar gene signatures for acute rejection are also indicative of CKTR. For example, a study by Khatri et al. (85) revealed 11 genes associated with acute rejection across different engrafted tissues, among which 7 genes (*CD6, INPP5D, ISG20, NKG7, PSMB9, RUNX3*, and *TAP1*) were identified as predictors for the development of progressive i-IFTA at 24 months posttransplant (45). More interestingly, a set of four gene markers (*vimentin, NKCC2, E-cadherin, and 18S rRNA*) in urine samples has been identified as reliable non-invasive biomarkers for i-IFTA (46).

### Epigenetic Biomarkers

Epigenetic modifications and regulators control relevant gene expression and function in response to altered biological process, and thereby can be employed as disease biomarkers (86). Epigenetic modifications include cytosine methylation of DNA at cytosine-phosphate diester-guanine dinucleotides, microRNA interactions, histone modifications, and chromatin remodeling complexes (87), which occur to genome without alteration of the DNA sequence. Epigenetics is an emerging field of research in kidney transplantation. Most studies have been performed in the context of ischemia and reperfusion injury and acute rejection, demonstrating the implication of aberrant DNA methylation (88). Recent studies in both humans and animals (54, 89) have shown that altered epigenetic modifications, particularly DNA methylation, influences the activation, proliferation, differentiation, and migration of a variety of cell types, e.g. helper T cells (90, 91) or regulatory T cells (54) and fibroblast (92), which are implicated in allograft survival and kidney fibrosis. For example, Foxp3 demethylation at the T(reg)-specific demethylation region positively correlates with numbers of intragraft Foxp3-expressing T cells in patients with subclinical rejection with i-IFTA *via* protocol biopsies; consequently, patients with more Foxp3+ T(reg) cells within graft infiltrates showed significantly better 5-year graft function evolution than patients without Foxp3+ T(reg) cell infiltration (54). Boer et al. (55) studied DNA methylation (DNAm) of the pro-inflammatory cytokine interferon γ (IFNγ) and the inhibitory receptor programmed death 1 (PD1) in naïve and memory CD8+ T cell subsets in kidney transplant recipients. Increased DNAm of IFN-γ and PD1 was observed in memory CD8+ T cells in kidney transplant recipients 3 months after transplantation, regardless of a rejection episode or not, suggesting that it was a non-specific change associated with transplant surgery or use of immunosuppressive drugs. However, PD1 methylation in the CD27− memory CD8+ T cells was more prominently increased in recipients with rejection episode than those without. In a more recent study concerning the role of DNAm in progression of IFTA in renal biopsies, normal allograft biopsies at 2-years post-transplantation showed similar DNAm patterns comparable to preimplantation biopsies, whereas persistent differentially methylation was associated with progression of allografts to chronic renal allograft dysfunction (93). Epigenetic mechanisms such as hypomethylation could directly boost and indirectly modulate their expression by controlling miRNAs (93). Recent studies have revealed that mi-R21 and miR200b expression in urine are associated with IFTA and CAD (56), while circulating miR-150, miR192, miR-200b, and miR-423-3p in plasma are related to IFTA (57). Meanwhile, expression of miR21, miR-155, and miR-142-3p was up-regulated in the plasma of patients with IFTA (58), while miR-145-5p and miR-148a were down-regulated (59, 60). Another study showed that expression of miR-142-3p was up-regulated, whereas miR-204 and miR-211 were down-regulated both in urine and kidney graft of recipients with CAD-IFTA (61). In addition, up-regulation of miR142-5p, and down-regulation of miR-486-5p may serve as biomarkers for early detection of chronic ABMR (62). These markers could, therefore, be considered as potential markers for CAD.

### Proteomic Biomarkers

Scores of non-invasive proteomic biomarkers of CKTR are generated using high-throughput proteomic techniques, such as liquid chromatography-mass spectrometry (LC-MS), isobaric tag for relative and absolute quantitation (iTRAQ), protein microarray, and bead-based immunoassay. Studies investigating non-invasive proteomic biomarkers in urine and blood (94), have discovered unique protein sets valuable for differential diagnosis. For example, one study on a set of 245 urine samples from a pediatric and young adult kidney allograft recipient cohort, identified 35 proteins that could discriminate three types of graft injury, 11 peptides for acute rejection, 12 urinary peptides for chronic allograft nephropathy and 12 peptides for BK virus nephritis (63). Metzger et al. (95) validated a multi-marker urinary peptide classifier constructed from capillary electrophoresis mass spectrometry (CE-MS) peptide spectra of urine from a training set of 39 allograft patients to discriminate TCMR from healthy allografts. Srivastava et al. (64, 65) identified that the up-expression of urine ANXA11, Integrin α3, Integrin β3 and TNF-α, and the downregulation of serum PARP1 could be used as candidate proteomic biomarkers for kidney allograft rejection. Furthermore, several proteins, some chemokines and cytokines in blood and urine are also identified as biomarkers for diagnosing CKTR and predicting graft outcomes (66–71). Several recent efforts have established urinary C-X-C motif chemokine 9 (CXCL9) and CXCL10 as reliable biomarkers for subclinical allograft rejection and for guiding the post-transplant management (66, 67). A recent study shows that platelets contain a wide array of mediators that could potentially promote acute and chronic ABMR (96, 97). In fact, platelet factor 4 (PF4, also known as CXCL4), the most abundant platelet-related mediator detected in the allograft with large quantities, has multiple consequences on allografts, one of which is to promote monocytes survival and macrophage differentiation (98), predicting poorer graft outcomes (99).

### Metabolomic Biomarkers

Metabolomics is a rapidly emerging research field that involves comprehensive analysis of all metabolites in a single biological sample (100) and has recently gained tremendous interest in the biomarker study in organ transplantation. Compared to proteomic or transcriptomic markers, metabolomic biomarkers may be more precise in reflecting cellular functions (101). Metabolomics can be used in two ways: intensively analyzing and identifying individual metabolites; or using pattern recognition to record spectral patterns and intensities instead of recording individual molecules (100, 102). Researchers recommend that metabolomic markers improve observing rejection and other organ injuries (103). In children, urinary metabolomics improved detection of borderline TCMR and demonstrated promise in ABMR (104). Measuring adenosine triphosphate (ATP) generation by mitogen-stimulated CD4 lymphocytes (ImmuKnow assay) is an FDA-approved biomarker potentially effective in transplant recipients (72). In a randomized prospective study, based on immune function values determined by ImmuKnow assay, one-year patient survival was markedly improved and infection rates were reduced in the group receiving ATP release biomarker-guided immunosuppressant regulation (105). In a recent study, a panel of nine differential metabolites in urine were identified as novel potential metabolite biomarkers of TCMR (73). The metabolomic biomarkers that considered as potential markers for rejection episodes are listed in **Table 1** (72–78).

### Cellular Biomarkers

There has been significant attentions drawn to quantify alloreactive CD8+ T cells as potential cellular biomarkers of rejection (25, 79, 106), or tolerance (107). Ashokkumar et al. (80) found that allospecific CD154+ T-cytotoxic memory cells were associated with rejection risk in liver transplant recipients. Limited data showed that an increase in CD154+ subset is implicated in acute kidney transplant rejection (81). Resent studies showed that monitoring alloreactive memory IFN-γ-producing T cells could assess subclinical TCMR and predict *de novo* DSA (82), while ratio of T follicular helper cells and T follicular regulatory cells (Tfc/Tfr) was an independent risk factor for CAD (83). However, multicenter validation of its diagnostic/prognostic biomarker utility in CKTR remains to be determined (108). Both macrophages and NK cells are implicated in chronic rejection (21, 109–111). However, it remains to be determined whether a specific subset of macrophages or NK cells could be served as cellular markers for CKTR. Recently, single-cell sequencing technologies have been rapidly developed and have evolved as a power tool for unbiased assessments of genomic, epigenomic, and transcriptomic profiling at the single-cell level. Compared with traditional sequencing technology, single-cell technologies have the advantages of detecting heterogeneity among individual cells, distinguishing a small number of cells, and delineating cell maps (112, 113). Using scRNA-seq technique, Liu et al. revealed multiple novel subsets of immune cells, including five subclasses of NKT cells, two subtypes in memory B cells, a classic CD14+ group and a nonclassical CD16+ group in monocytes, in patients with CKTR. They also identified a novel subpopulation [myofibroblasts (MyoF)] in fibroblasts, which express collagen and extracellular matrix components in CKTR group (84). While still in its early infancy, scRNA-seq is considered as diagnostic tool for identifying cellular and molecular biomarkers specific for CKTR. With improved understanding of cellular mechanisms underlying CKTR and advances in the multi-color flow cytometry analyses combining with more recent development of single-cell genomics studies, it is conceivable that more precise cellular biomarkers will be identified for CKTR.

Several considerations ought to be adequately addressed before these biomarkers can be regularly used in the clinical practice for kidney transplants (114–116). First, sensitivity, specificity, positive and negative predictive values must be considered, and receiver operating characteristic (ROC) curves need to be thoroughly assessed for their clinical utility. Secondly, integration of different biomarkers is necessary for accurate diagnosis. Thirdly, robust validation studies and standardization of measurements are required to identify new biomarkers. Finally, timing required for generating results and cost of assessment should be reasonable.

## New Therapies for the Treatment of CKTR

Chronic active ABMR is the most widely recognized cause of allograft failure (117), whereas TCMR usually exists in a mixed rejection phenotype (118). Given current understanding that that chronic active TCMR is often associated with insufficient immunosuppression, TCMR treatment has been directed to increasing doses and types of anti-T cell immunosuppressive agents such as combinations of therapies with basiliximab, everolimus in addition to tacrolimus (119). Numerous therapies have been used in the clinical setting, mostly focusing on chronic active ABMR. The strategies include plasmapheresis, intravenous immunoglobulin (IVIG), CD20 antibody (rituximab), proteasome inhibitor (bortezomib) (120–122) and anti-complement monoclonal antibody (eculizumab), single or combined therapies (123, 124). Their therapeutic effectiveness in treatment of chronic active ABMR have been evaluated in recent randomized controlled trials and results have been extensively reviewed (125), suggesting limited success being achieved by using these agents alone or in combination despite their effectiveness in treating acute ABMR. Through biomarker discovery, understanding of CKTR has been tremendously improved over the last five years. Recognition of biological similarities shared by CKTR, cancer immunology and autoimmune diseases has led to frontier investigations in repurposing of several treatment strategies from cancer therapy or autoimmune diseases to ABMR. IL-6/IL-6R blockade (Tocilizumab), C1 esterase inhibitor (C1 INH), and B-lymphocyte stimulator (BLyS) inhibitor (Belimumab) are among those that have been tested for their therapeutic potentials in mitigating ABMR and have shown promising results as described below and summarized in **Table 2**.

**Table 2:** Clinical trials - new therapies for chronic ABMR after kidney transplantation.

Trial design	Inclusion criteria	Test therapeutics	Other Immuno suppression	Patients	Follow up	Major results	Ref
single center, open-label case study, historical control	chronic ABMR, DSA+, TG	**Tocilizumab** (8 mg/kg monthly, maximal dose 800 mg for 6–25 months)	Tac/MMF/Pred	36	6 years	reduction in DSAs and stabilization of renal function at 2 years; graft survival rate of 80%, patient survival rate of 91% at 6 years	Choi J, et al. (126)
randomized controlled trials	ABMR, DSA+	**C1 INH** (5000 U on day 1 of ABMR, 2500 U on days 3, 5, 7, 9, 11, and 13) add-on standard of care (PP+IVIG+/- anti‐CD20)	n/a	18 (treatment: n=9; placebo: n=9)	6 months	reduction of transplant glomerulopathy	Montgomery RA, et al. (127)
single center, observational study, historical control	refractory active ABMR with acute allograft dysfunction, DSA>3000 MFI, g+ptc≥2	**C1 INH** (20 units/kg on days 1, 2, and 3 and then twice weekly; IVIG at 2 g/kg every month for 6 months	Tac/MMF/Pred	6	6 months	improvement in eGFR, reduced DSA; no change in histological features	Viglietti D, et al. (128)
randomized controlled trials	adult patient receiving a kidney transplant	**Belimumab** (10 mg/kg on day 0, 14, and 28, and then every 4 weeks for a total of 7 infusions)	Tac/MMF/Pred	28 (treatment: n=14; placebo: n=14)	6 months	similar proportions of adverse events; no change in the number of naive B cells	Banham GD, et al. (129)

TG, transplant glomerulopathy; Tac, tacrolimus; MMF, mycophenolate mofetil; Pred, prednisone; n/a, not available.

### IL-6/IL-6R Blockade

IL-6 is a pleotropic cytokine associated to many facets of innate and adaptive immunity, which plays an important role in DSA generation and chronic ABMR, including its effects on B cell immunity and antibody-producing plasma cells, as well as the balance between effector and regulatory T cells (130). Blockade of the IL-6/IL-6R axis with Tocilizumab, anti–interleukin-6 receptor monoclonal antibody has been well-established for the treatment of rheumatoid arthritis (131), and is recently considered as a new therapy to prevent ABMR progression (126). It has been shown that tocilizumab markedly reduced DSAs and stabilized renal function at 2 years post-transplant, suggesting a therapeutic effect of tocilizumab in ABMR. Tocilizumab has also been evaluated in combination with IVIG and rituximab for patients who failed standard desensitization, and it appeared well tolerated and safe (132). However, there is still a lack of randomized controlled trials to systematically evaluate the efficacy and safety of tocilizumab to date. Another new inhibitor for IL-6/IL-6R axis is clazakizumab, a genetically engineered humanized monoclonal antibody directed against IL-6. Two pilot trials (NCT03444103, NCT03380377) (132–134) and a large multicenter trial evaluating clazakizumab in late/chronic ABMR (NCT03744910) (135) are underway.

### C1 Esterase Inhibitor (C1 INH)

Since the efficacy of C5 blockade in late ABMR is limited (123, 124), blockade of early complement pathway at the level of key component C1 has attracted a great deal of attention. One potential strategy being studied is the use of C1 INH, which has been used to prevent and/or treat attacks of hereditary angioedema for years and has an established safety record (136). C1-INH is a serum protease inhibitor that binds covalently and inactivates C1r, C1s, and mannan-binding protein-associated proteases (136, 137). In a double-blind RCT, C1-INH was tested as a treatment for biopsy-proven ABMR. Both C1-INH and placebo groups showed improvements in early follow-up biopsies. However, in a subset of patients with late follow-up biopsies (6 months), a decreased rate of transplant glomerulopathy was seen in C1-INH treated group, accompanied by improved graft function, suggesting C1-NIH may be effective in preventing the development of chronic injury (127). In a prospective, single-arm pilot clinical trail, C1-INH was added to IVIG to treat refractory acute ABMR. In comparison with historical controls, patients treated with C1-INH showed decreased C4d deposition and improved renal function, whereas microcirculatory damage still persisted (glomerulitis, peritubular capillaritis, and allograft glomerulopathy) (128). Currently, a large multicenter clinical trials evaluating C1-INH added to standard treatment of ABMR (NCT02547220) (138) is underway, while another clinical trial evaluating C1-NIH for the treatment of refractory AMR (NCT03221842) in renal transplant recipients (139) is also ongoing.

### Inhibition of B-lymphocyte Stimulator

B-lymphocyte stimulator (BLyS) is a critical cytokine that enhances B cell and plasma cell survival (140). Targeting BLyS has recently driven increasing interest in transplant by modulating B cell alloimmunity. Belimumab, a humanized anti-BLyS antibody, which has shown therapeutic efficacy in systemic lupus erythematosus (141), has now been applied in organ transplantation. In a double-blind, randomized, placebo-controlled phase 2 trial, belimumab was evaluated in 28 kidney transplant recipients (129). The findings revealed that treatment of belimumab showed no effect on reducing the number of naïve B cells from baseline to 24 weeks after transplant. However, the activated memory B cells and plasmablasts were significantly reduced, and tissue-specific antibodies in serum were lowered. In addition, treatment with belimumab modulated the B cell profile towards a regulatory profile by changing the IL-10/IL-6 ratio. In parallel, genes coding for IgG and markers of T cell proliferation were reduced (129). To date, there is still lack of clinical trial using belimumab to treat chronic rejection. In a murine chronic ABMR kidney transplant model, blockade of APRIL/BLyS by TAC-Ig resulted in decreased antinuclear antibody (ANA) and disruption of splenic germinal center architecture, but have no significant difference in lymphocyte infiltration and kidney graft pathology compared with control grafts, which may be due to the absence of T cell immunosuppression (142).

## Conclusion

Discovery of novel earlier diagnostic biomarkers will not only allows designing individualized therapy for timely therapeutic intervention, but also further advance understanding of pathogenesis of CKTR. Although many biomarkers listed in **Table 1** still require validation and standardization in several independent cohorts, considerable progress has been made in recent years (115, 116, 143). The management of CKTR remains a daunting task due to the complex pathogenesis of CKTR and irreversibility at the time of diagnosis. Nevertheless, several promising therapies have been in robust intervention trials with promising results. With the emergence of new technologies, such as single cell genomics, computational biology along with artificial intelligence-based assistance, it is conceivable that more specific biomarkers and therapeutic targets for CKTR will be identified and translated into the clinical practice in the very near future.

## Author Contributions

XL and XZ: participated in manuscript preparation and writing. JM, LG, and JL: provided suggestion and edits. ZZ conceptualized, wrote, and revised manuscript All authors contributed to the article and approved the submitted version.

## Conflict of Interest

The authors declare that the research was conducted in the absence of any commercial or financial relationships that could be construed as a potential conflict of interest.
